# Oblique stagnation point flow of magnetized Maxwell fluid over a stretchable Riga plate with Cattaneo-Christov heat flux and convective conditions

**DOI:** 10.1038/s41598-023-35263-9

**Published:** 2023-09-29

**Authors:** Mirza Naveed Jahangeer Baig, Nadeem Salamat, Salman Akhtar, Sohail Nadeem

**Affiliations:** 1 Institute of Mathematics, Khawaja Fareed University of Engineering Information and Technology, Rahim Yar Khan, 64200 Pakistan; 2 Department of Mathematics, Quaid-I-Azam University, Islamabad, Pakistan; 3 Department of Mathematics, Wenzhou University, Wenzhou, 325035 China

**Keywords:** Engineering, Materials science, Mathematics and computing, Physics

## Abstract

The current work deals with the oblique stagnation point flow phenomenon of a rate-type Maxwell fluid with the significance of the Cattaneo-Christov double diffusion theory. The Cattaneo-Christov theory is illustrated through the modified form of Fourier’s and Fick’s laws. The steady magnetized flow mechanism is observed in two dimensions through a stretchable convective Riga plate. In the mass and heat transfer analysis, the consequences of chemical reactions and thermal radiation are also incorporated. With the contribution of relevant dimensionless quantities, the setup of dimensionless equations is acquired which further takes the form of nonlinear equations. The physical significance of the numerous parameters on different features of the flow phenomenon is graphically exhibited. The interesting physical quantities are computed and numerically evaluated relative to the pertinent parameters. This study reveals that the thermal relaxation time parameter lowers the rate of heat transfer, and the thermal Biot number enhances the rate of heat transport. Moreover, the Deborah number minimizes the flow field of both tangential and axial velocities.

## Introduction

Due to the numerous utilizations of stagnation point flows in different areas, the significance of such flows is enhanced. The applications of such flows include adhesive materials manufacturing, artificial fibers production, industrial problems, electrical device cooling, and paper drying^1^. A flow in which the fluid obliquely imposes on the surface and makes an acute angle is identified as the oblique stagnation point flow. Such flows have been investigated by numerous researchers with the involvement of different physical effects. With the involvement of a stretchable medium, Ghaffari et al.^2^ scrutinized the oblique stagnant point flow mechanism of Walter’s fluid influenced by thermal radiation. They observed an increment in the rate of heat transfer with the improved Biot number. The time-independent flow phenomenon of a micropolar fluid near an oblique stagnation point with the impact of partial slip was addressed by Lok et al.^3^. They performed a stability analysis of the flow problem and acquired dual results. Nadeem and Khan^4^ discussed the dual nature of magnetized nanofluid flow generated by a stretchable oscillatory medium near an oblique stagnant point. They examined that the stronger magnetic field augmented the physical quantities. Abbasi et al.^5^ demonstrate the flow mechanism and thermal characteristics of a non-Newtonian Maxwell fluid through an oblique stagnant point relative to a stretched convective medium. They examined that in the case of the non-convective medium, the fields of concentration and temperature are larger in contrast to the convective medium. More investigations related to oblique stagnation point flow with the contribution of numerous physical properties are discussed in Refs.^6–11^.

Due to the significant rheological characteristics of non-Newtonian fluids, such fluids exhibit many practical applications such as metal spinning, food processing, optical fibers, extrusion of polymers, plastic polymer production, etc. These fluids are very complicated in their nature and cannot be defined through an individual equation. To deliberate all the characteristics of such fluids, numerous model has been introduced. Among them, one of the significant models is the rate-type Maxwell fluid. The Maxwell fluid model has grabbed the attention of various researchers due to the effects of relaxation time. Through a porous medium, Wang and Tan^12^ studied the time-dependent flow mechanism of a viscoelastic Maxwell fluid with the contribution of physical effect. They performed stability analysis through linear and nonlinear theories and examined that the system’s instability intensifies with the relaxation time parameter. Ramzan et al.^13^ scrutinized the thermal features and three-dimensional flow phenomenon influenced by various physical effects in a Maxwell fluid. They observed a higher rate of heat transfer with a greater amount of heat source. A scrutinization of the chemically reactive radiative Maxwell fluid flow problem subject to a convective porous medium was conducted by Hosseinzadeh et al.^14^. They conclude that the higher magnitude of the radiation parameter yields the increasing behavior of the thermal field. Through a stretchable surface, Kumar et al.^15^ numerically analyze the two-dimensional time-independent flow phenomenon of a Maxwell fluid conveying nanoparticles with the significance of the magnetic dipole. Abdal et al.^16^ studied the bioconvection radiative flow problem of a non-Newtonian Maxwell fluid with the significance of the physical boundary conditions. They observed the higher heat transfer rate of the Maxwell fluid due to the escalating nature of the thermal radiation parameter. With the involvement of the two-phase Buongiorno model, the radiative flow mechanism generated by two stretchable disks in a Maxwell fluid was addressed by Chu et al.^17^.

The heat transport phenomenon developed between two objects can be described through conventional theories. Fourier^18^ initially discussed the heat transfer mechanism through Fourier’s law of heat conduction without the involvement of time. After that, with the contribution of thermal relaxation time, the modified form of Fourier’s law was proposed by Cattaneo^19^. The theory of Cattaneo^19^ was further modified by Christov^20^. Afterward, to explore the heat transfer mechanism, the Cattaneo-Christov theory was introduced. Numerous researchers observe the thermal properties of physical problems with the collaboration of Cattaneo-Christov theory. Chu et al.^21^ worked on a second-grade fluid to discuss the Cattaneo-Christov theory on heat and mass transport mechanisms. They acquired the analytical results of the flow problem and physically explored the fluid properties. The study of the thermal features of a water-based nanofluid with the contribution of the Cattaneo-Christov model was disclosed by Rawat et al.^22^. They analyzed the mechanism of mass and heat transport through the effects of chemical reaction and thermal radiation respectively. Moreover, a reduction in the magnitude of concentration and temperature curves was found corresponding to the escalating solutal and relaxation time parameters respectively. Through a stretchable curved surface, Madhukesh et al.^23^ explored the thermal mechanism of a hybrid nanofluid with the involvement of heat flux theory. Rasool et al.^24^ considered a porous surface to analytically scrutinize the mass and thermal features of second grade nanofluid with the significance of the Cattaneo-Christov theories and various physical effects.

The branch of magneto hydrodynamics is concerned with the behavior of electrically conducting fluid with the presence of magnetic characteristics. With the various implementations of MHD flows such as heat exchangers, filtration, power pumps, and medical sciences, such flows have become essential for researchers. The axisymmetric magnetized flow mechanism and heat transport phenomenon influenced by the thermal radiation of a nanofluid was inspected by Jyothi et al.^25^. A reduction in the flow field with the improved magnetic field was observed in their study. Through a stretched vertical medium, Ramamoorthy and Pallavarapu^26^ explored the three-dimensional hydromagnetic flow of a Willamson fluid with the consequence of the chemical reaction and thermal radiation. They obtained the results of the flow phenomenon through numerical technique and physically visualized the impacts of the pertinent parameters on the flow system. An investigation of the magnetized time-independent flow phenomenon developed by a shrinking/stretchable surface in a Maxwell fluid through a stagnation point was disclosed by Khan et al.^27^. An exploration of the time-dependent flow problem of a Maxwell nanofluid with the significance of the variable physical property and magnetic field was investigated by Islam et al.^28^. They conclude that the reduction rate of the velocity field relative to the larger magnetic field is high for the cylindrical medium as compared to the sheet.

After analyzing the aforementioned literature review, we observe that the oblique stagnation point flow of Maxwell fluid due to a stretchable sheet has been studied but the oblique stagnation point flow of Maxwell fluid with the presence of Cattaneo-Christov double diffusion theory and chemical reaction over a stretchable convective Riga plate has not been examined until now. The current flow problem has the novelty of the two-dimensional steady oblique stagnant point flow behavior of a Maxwell fluid over a convective surface of a stretchable Riga plate. Moreover, the heat and mass transfer mechanisms are investigated through the implementation of heat and mass flux theories with the effects of thermal radiation and chemical reaction. The setup of dimensionless equations is obtained after the execution of the non-dimensional variables. These dimensionless equations are further transformed into a nonlinear system of ordinary differential equations. A physical visualization of different features of flow phenomenon (concentration, velocity, temperature) corresponding to the pertinent parameters is discussed through graphs. The current paper is organized in the following pattern, the problem’s mathematical formulation is described in Section “Problem’s formulation”, Section “Result and explanation” deals with the results and explanations of the flow problem, and the purpose of Section “Concluding remarks” is to conclude the main findings of the problem.

## Problem’s formulation

**Figure 1:**
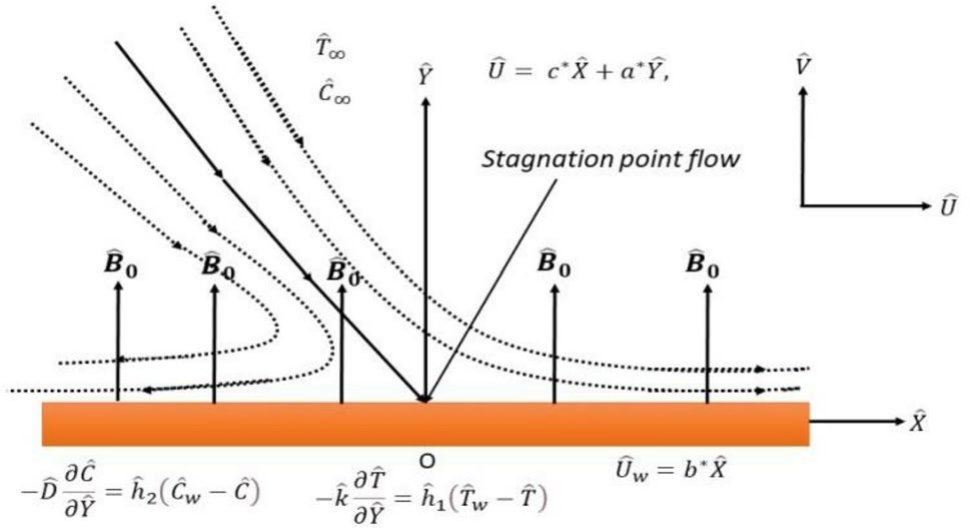
Flow problem’s geometrical configuration.

Let us take a Riga stretchable plate to scrutinize the two-dimensional steady flow of a Maxwell fluid. In the Cartesian coordinates system, the Riga plate is taken along the $\widehat{X}$-axis and assumed to be stretched with the velocity ${\widehat{U}}_{w}={b}^{*}\widehat{X}, \left({b}^{*}>0\right)$ through the implementation of two opposite and equal forces. The direction of $\widehat{Y}$-axis is normal to the $\widehat{X}$-axis, as shown in Fig. 1. The electrically conducting fluid is obliquely impinging on a convective Riga plate and is considered to flow in the region of $\widehat{Y}>0$. A magnetic field with uniform strength ${\widehat{B}}_{0}$ is executed towards the $\widehat{Y}$-axis. Moreover, the effect of thermal radiation with Rosseland approximation is involved in the thermal analysis.

For the current problem, the constituting equations can be written in vector form as follows^5^,

1 $$\nabla .{\varvec{V}}=0,$$

2 $$\widehat{\rho }\left(\frac{\partial {\varvec{V}}}{\partial \widehat{t}}+\left({\varvec{V}}.\nabla \right){\varvec{V}}\right)=-\nabla \widehat{P}+\nabla .\overline{{\varvec{\tau}} }+\widehat{\sigma }\left({\varvec{V}}\times {\varvec{B}}\right)\times {\varvec{B}},$$

3 $$\widehat{\rho }{\widehat{C}}_{\widehat{P}}\left(\frac{\partial \widehat{T}}{\partial \widehat{t}}+\left({\varvec{V}}.\nabla \right)\widehat{T}\right)=-\nabla .\mathbf{q},$$

4 $$\left(\frac{\partial \widehat{C}}{\partial \widehat{t}}+\left({\varvec{V}}.\nabla \right)\widehat{C}\right)=-\nabla .\mathbf{J},$$

For the Maxwell fluid, the extra stress tensor has the following expressions^5^,

5 $$\overline{{\varvec{\tau}} }+\widehat{\lambda }\frac{D\overline{{\varvec{\tau}}}}{D\widehat{t} }=\widehat{\mu }\overline{{\varvec{A}} },$$

The contravariant vector and first Rivlin-Ericksen tensor have the following form^5^,

6 $$\frac{D\overline{{\varvec{\tau}}}}{D\widehat{t} }=\frac{d\overline{{\varvec{\tau}}}}{d\widehat{t} }-\left(\nabla {\varvec{V}}\right)\overline{{\varvec{\tau}} }-\overline{{\varvec{\tau}} }{\left(\nabla {\varvec{V}}\right)}^{t},\boldsymbol{ }\boldsymbol{ }\overline{{\varvec{A}} }=\nabla {\varvec{V}}+{\left(\nabla {\varvec{V}}\right)}^{t},$$

In Eq. (3), we have implemented the Cattaneo-Christov theory for heat and mass fluxes as follows^29^,

7 $$\mathbf{q}+{\widehat{\gamma }}_{E}\left({\mathbf{q}}_{\widehat{t}}+\left(\nabla . \mathbf{V}\right)\mathbf{q}+\mathbf{V} .\nabla \mathbf{q}-\mathbf{q}.\nabla \mathbf{V}\right)= -\widehat{k}\nabla \widehat{T},$$

8 $$\mathbf{J}+{\widehat{\gamma }}_{C}\left({\mathbf{J}}_{\widehat{t}}+\left(\nabla . \mathbf{V}\right)\mathbf{J}+\mathbf{V} .\nabla \mathbf{J}-\mathbf{J}.\nabla \mathbf{V}\right)= -\widehat{D}\nabla \widehat{C},$$

Equations (7) and (8) yield the fundamental Fourier and Ficks laws with ${\widehat{\gamma }}_{E}={\widehat{\gamma }}_{C}=0$. As, we have to deal with a steady and incompressible fluid. So, we get the following form of Eqs. (7) and (8).

9 $$\mathbf{q}+{\widehat{\gamma }}_{E}\left(\mathbf{V} .\nabla \mathbf{q}-\mathbf{q}.\nabla \mathbf{V}\right)= -\widehat{k}\nabla \widehat{T},$$

10 $$\mathbf{J}+{\widehat{\gamma }}_{C}\left(\mathbf{V} .\nabla \mathbf{J}-\mathbf{J}.\nabla \mathbf{V}\right)= -\widehat{D}\nabla \widehat{C},$$

For the ongoing two-dimensional flow problem, the velocity vector has the following components,

11 $$\mathbf{V}=\left(\widehat{U},\widehat{V}\right)=\left(\widehat{U}\left(\widehat{X},\widehat{Y}\right), \widehat{V}\left(\widehat{X},\widehat{Y}\right)\right),$$

According to Eqs. (1), (2), (3), (4), (5), (6), (7), (8), (9), (10) and (11), the current flow problem has the following equations^2,5,29–31^

12 $$\frac{\partial \widehat{U}}{\partial \widehat{X}}+\frac{\partial \widehat{V}}{\partial \widehat{Y}}=0,$$

$$\widehat{U}\frac{\partial \widehat{U}}{\partial \widehat{X}}+\widehat{V}\frac{\partial \widehat{U}}{\partial \widehat{Y}}=-\frac{1}{\widehat{\rho }}\frac{\partial \widehat{P}}{\partial \widehat{X}}+\widehat{\vartheta }\left(\frac{{\partial }^{2}\widehat{U}}{\partial {\widehat{X}}^{2}}+\frac{{\partial }^{2}\widehat{U}}{\partial {\widehat{Y}}^{2}}\right)+\frac{\widehat{\lambda }}{\widehat{\rho }}\left(\widehat{U}\frac{{\partial }^{2}\widehat{P}}{\partial {\widehat{X}}^{2}}+\widehat{U}\frac{{\partial }^{2}\widehat{P}}{\partial \widehat{X}\partial \widehat{Y}}-\frac{\partial \widehat{U}}{\partial \widehat{X}}\frac{\partial \widehat{P}}{\partial \widehat{X}}-\frac{\partial \widehat{U}}{\partial \widehat{Y}}\frac{\partial \widehat{P}}{\partial \widehat{Y}}\right)$$

13 $$- \widehat{\lambda }\left(2\widehat{U}\widehat{V}\frac{{\partial }^{2}\widehat{U}}{\partial \widehat{X}\partial \widehat{Y}}+{\widehat{V}}^{2}\frac{{\partial }^{2}\widehat{U}}{\partial {\widehat{Y}}^{2}}+{\widehat{U}}^{2}\frac{{\partial }^{2}\widehat{U}}{\partial {\widehat{X}}^{2}}\right)-\frac{\widehat{\sigma }}{\widehat{\rho }}{\widehat{B}}_{0}^{2}\widehat{U}+\frac{\pi {\widehat{j}}_{0}{\widehat{M}}_{0}}{8\widehat{\rho }}\mathrm{exp}\left(-\left(\frac{\pi }{\widehat{\delta }}\right)\widehat{Y}\right),$$

$$\widehat{U}\frac{\partial \widehat{V}}{\partial \widehat{X}}+\widehat{V}\frac{\partial \widehat{V}}{\partial \widehat{Y}}=-\frac{1}{\widehat{\rho }}\frac{\partial \widehat{P}}{\partial \widehat{Y}}+\widehat{\vartheta }\left(\frac{{\partial }^{2}\widehat{V}}{\partial {\widehat{X}}^{2}}+\frac{{\partial }^{2}\widehat{V}}{\partial {\widehat{Y}}^{2}}\right)+\frac{\widehat{\lambda }}{\widehat{\rho }}\left(\widehat{V}\frac{{\partial }^{2}\widehat{P}}{\partial {\widehat{Y}}^{2}}+\widehat{V}\frac{{\partial }^{2}\widehat{P}}{\partial \widehat{X}\partial \widehat{Y}}-\frac{\partial \widehat{V}}{\partial \widehat{X}}\frac{\partial \widehat{P}}{\partial \widehat{X}}-\frac{\partial \widehat{V}}{\partial \widehat{Y}}\frac{\partial \widehat{P}}{\partial \widehat{Y}}\right)$$

14 $$- \widehat{\lambda }\left(2\widehat{U}\widehat{V}\frac{{\partial }^{2}\widehat{V}}{\partial \widehat{X}\partial \widehat{Y}}+{\widehat{V}}^{2}\frac{{\partial }^{2}\widehat{V}}{\partial {\widehat{Y}}^{2}}+{\widehat{U}}^{2}\frac{{\partial }^{2}\widehat{V}}{\partial {\widehat{X}}^{2}}\right),$$

15 $$\widehat{U}\frac{\partial \widehat{T}}{\partial \widehat{X}}+\widehat{V}\frac{\partial \widehat{T}}{\partial \widehat{Y}}+{\widehat{\gamma }}_{E}{\widehat{\Phi }}_{E}=\frac{\widehat{k}}{\widehat{\rho }{\widehat{C}}_{\widehat{P}}}\left(\frac{{\partial }^{2}\widehat{T}}{\partial {\widehat{X}}^{2}}+\frac{{\partial }^{2}\widehat{T}}{\partial {\widehat{Y}}^{2}}\right)-\frac{1}{\widehat{\rho }{\widehat{C}}_{\widehat{P}}}\left(\frac{\partial {\widehat{q}}_{r}}{\partial \widehat{Y}}+\frac{\partial {\widehat{q}}_{r}}{\partial \widehat{X}}\right),$$

16 $$\widehat{U}\frac{\partial \widehat{C}}{\partial \widehat{X}}+\widehat{V}\frac{\partial \widehat{C}}{\partial \widehat{Y}}+{\widehat{\gamma }}_{C}{\widehat{\Phi }}_{C}=\widehat{D}\left(\frac{{\partial }^{2}\widehat{C}}{\partial {\widehat{X}}^{2}}+\frac{{\partial }^{2}\widehat{C}}{\partial {\widehat{Y}}^{2}}\right)-\widehat{\xi }\left(\widehat{C}-{\widehat{C}}_{\infty }\right),$$

Here

17 $${\widehat{\Phi }}_{E}={\widehat{U}}^{2}\frac{{\partial }^{2}\widehat{T}}{\partial {\widehat{X}}^{2}}+\widehat{U}\frac{\partial \widehat{T}}{\partial \widehat{X}}\frac{\partial \widehat{U}}{\partial \widehat{X}}+\widehat{U}\frac{\partial \widehat{V}}{\partial \widehat{X}}\frac{\partial \widehat{T}}{\partial \widehat{Y}}+2\widehat{U}\widehat{V}\frac{{\partial }^{2}\widehat{T}}{\partial \widehat{X}\partial \widehat{Y}}+{\widehat{V}}^{2}\frac{{\partial }^{2}\widehat{T}}{\partial {\widehat{Y}}^{2}}+\widehat{V}\frac{\partial \widehat{T}}{\partial \widehat{Y}}\frac{\partial \widehat{V}}{\partial \widehat{Y}}+\widehat{V}\frac{\partial \widehat{U}}{\partial \widehat{Y}}\frac{\partial \widehat{T}}{\partial \widehat{X}},$$

18 $${\widehat{\Phi }}_{C}={\widehat{U}}^{2}\frac{{\partial }^{2}\widehat{C}}{\partial {\widehat{X}}^{2}}+\widehat{U}\frac{\partial \widehat{C}}{\partial \widehat{X}}\frac{\partial \widehat{U}}{\partial \widehat{X}}+\widehat{U}\frac{\partial \widehat{V}}{\partial \widehat{X}}\frac{\partial \widehat{C}}{\partial \widehat{Y}}+2\widehat{U}\widehat{V}\frac{{\partial }^{2}\widehat{C}}{\partial \widehat{X}\partial \widehat{Y}}+{\widehat{V}}^{2}\frac{{\partial }^{2}\widehat{C}}{\partial {\widehat{Y}}^{2}}+\widehat{V}\frac{\partial \widehat{C}}{\partial \widehat{Y}}\frac{\partial \widehat{V}}{\partial \widehat{Y}}+\widehat{V}\frac{\partial \widehat{U}}{\partial \widehat{Y}}\frac{\partial \widehat{C}}{\partial \widehat{X}},$$

In view of the Rosseland approximation, the radiative heat flux in Eq. (15) has the following form^2^,

19 $${\widehat{q}}_{r}=-\frac{16{\sigma }_{1}{\widehat{T}}_{\infty }^{3}}{3{\widehat{k}}_{1}}\frac{\partial \widehat{T}}{\partial \widehat{Y}},$$

For the above system of equations, the boundary conditions are expressed as^5^

$$-\widehat{D}\frac{\partial \widehat{C}}{\partial \widehat{Y}}={\widehat{h}}_{2}\left({\widehat{C}}_{w}-\widehat{C}\right), \widehat{V}=0, -\widehat{k}\frac{\partial \widehat{T}}{\partial \widehat{Y}}={\widehat{h}}_{1}\left({\widehat{T}}_{w}-\widehat{T}\right), \widehat{U}= {\widehat{U}}_{w}={b}^{*}\widehat{X,} \mathrm{at} \widehat{Y}=0,$$

20 $$\widehat{C}\to {\widehat{C}}_{\infty }, \widehat{U}= {c}^{*}\widehat{X}+{a}^{*}\widehat{Y,} \widehat{T}\to {\widehat{T}}_{\infty } \mathrm{as} \widehat{Y}\to \infty .$$

To acquire the dimensionless form of the above setup of equations, we adopt the following dimensionless variables^5^,

$${C}^{*}={\left({\widehat{C}}_{w}-{\widehat{C}}_{\infty }\right)}^{-1}\left(\widehat{C}-{\widehat{C}}_{\infty }\right), {X}^{*}=\widehat{X}{\left(\frac{{b}^{*}}{\widehat{\vartheta }}\right)}^{1/2}, {V}^{*}=\widehat{V}{(\widehat{\vartheta }{b}^{*})}^{-1/2}, {P}^{*}=\frac{1}{\widehat{\vartheta }\widehat{\rho }{b}^{*}} ,$$

21 $${T}^{*}={\left({\widehat{T}}_{w}-{\widehat{T}}_{\infty }\right)}^{-1}\left(\widehat{T}-{\widehat{T}}_{\infty }\right), {U}^{*}=\widehat{U}{(\widehat{\vartheta }{b}^{*})}^{-1/2}, {Y}^{*}=\widehat{Y}{\left(\frac{{b}^{*}}{\widehat{\vartheta }}\right)}^{1/2}$$

The implementation of Eq. (21) to Eqs. (12), (13), (14), (15), (16), (17), (18) and (19) yield the following equations,

22 $$\frac{\partial {U}^{*}}{\partial {X}^{*}}+\frac{\partial {V}^{*}}{\partial {Y}^{*}}=0,$$

$${U}^{*}\frac{\partial {U}^{*}}{\partial {X}^{*}}+{V}^{*}\frac{\partial {U}^{*}}{\partial {Y}^{*}}=-\frac{\partial {P}^{*}}{\partial {X}^{*}}+\left(\frac{{\partial }^{2}{U}^{*}}{\partial {{X}^{*}}^{2}}+\frac{{\partial }^{2}{U}^{*}}{\partial {{Y}^{*}}^{2}}\right)+{\beta }^{*}\left({U}^{*}\frac{{\partial }^{2}{P}^{*}}{\partial {{X}^{*}}^{2}}+{U}^{*}\frac{{\partial }^{2}{P}^{*}}{\partial {X}^{*}\partial {Y}^{*}}-\frac{\partial {U}^{*}}{\partial {X}^{*}}\frac{\partial {P}^{*}}{\partial {X}^{*}}-\frac{\partial {U}^{*}}{\partial {Y}^{*}}\frac{\partial {P}^{*}}{\partial {Y}^{*}}\right)$$

23 $$- {\beta }^{*}\left(2{U}^{*}{V}^{*}\frac{{\partial }^{2}{U}^{*}}{\partial {X}^{*}\partial {Y}^{*}}+{{V}^{*}}^{2}\frac{{\partial }^{2}{U}^{*}}{\partial {{Y}^{*}}^{2}}+{{U}^{*}}^{2}\frac{{\partial }^{2}{U}^{*}}{\partial {{X}^{*}}^{2}}\right)-M{U}^{*}+A\mathrm{exp}\left(-\zeta {Y}^{*}\right),$$

$${U}^{*}\frac{\partial {V}^{*}}{\partial {X}^{*}}+{V}^{*}\frac{\partial {V}^{*}}{\partial {Y}^{*}}=-\frac{\partial {P}^{*}}{\partial {Y}^{*}}+\left(\frac{{\partial }^{2}{V}^{*}}{\partial {{X}^{*}}^{2}}+\frac{{\partial }^{2}{V}^{*}}{\partial {{Y}^{*}}^{2}}\right)+{\beta }^{*}\left({V}^{*}\frac{{\partial }^{2}{P}^{*}}{\partial {{Y}^{*}}^{2}}+{V}^{*}\frac{{\partial }^{2}{P}^{*}}{\partial {X}^{*}\partial {Y}^{*}}-\frac{\partial {V}^{*}}{\partial {X}^{*}}\frac{\partial {P}^{*}}{\partial {X}^{*}}-\frac{\partial {V}^{*}}{\partial {Y}^{*}}\frac{\partial {P}^{*}}{\partial {Y}^{*}}\right)$$

24 $$- {\beta }^{*}\left(2{U}^{*}{V}^{*}\frac{{\partial }^{2}{V}^{*}}{\partial {X}^{*}\partial {Y}^{*}}+{{V}^{*}}^{2}\frac{{\partial }^{2}{V}^{*}}{\partial {{Y}^{*}}^{2}}+{{U}^{*}}^{2}\frac{{\partial }^{2}{V}^{*}}{\partial {{X}^{*}}^{2}}\right),$$

$${U}^{*}\frac{\partial {T}^{*}}{\partial {X}^{*}}+{V}^{*}\frac{\partial {T}^{*}}{\partial {Y}^{*}}+{\delta }_{e}\left({{U}^{*}}^{2}\frac{{\partial }^{2}{T}^{*}}{\partial {{X}^{*}}^{2}}+{U}^{*}\frac{\partial {T}^{*}}{\partial {X}^{*}}\frac{\partial {U}^{*}}{\partial {X}^{*}}+{U}^{*}\frac{\partial {V}^{*}}{\partial {X}^{*}}\frac{\partial {T}^{*}}{\partial {Y}^{*}}+2{U}^{*}{V}^{*}\frac{{\partial }^{2}{T}^{*}}{\partial {X}^{*}\partial {Y}^{*}}+{{V}^{*}}^{2}\frac{{\partial }^{2}{T}^{*}}{\partial {{Y}^{*}}^{2}}\right.$$

25 $$\left.+{V}^{*}\frac{\partial {T}^{*}}{\partial {Y}^{*}}\frac{\partial {V}^{*}}{\partial {Y}^{*}}+{V}^{*}\frac{\partial {U}^{*}}{\partial {Y}^{*}}\frac{\partial {T}^{*}}{\partial {X}^{*}}\right)=\frac{1}{Pr}\left(\frac{{\partial }^{2}{T}^{*}}{\partial {{X}^{*}}^{2}}+\frac{{\partial }^{2}{T}^{*}}{\partial {{Y}^{*}}^{2}}\right)+\frac{4}{3}Rd\left(\frac{{\partial }^{2}{T}^{*}}{\partial {{Y}^{*}}^{2}}+\frac{{\partial }^{2}{T}^{*}}{\partial {X}^{*}\partial {Y}^{*}}\right),$$

$${U}^{*}\frac{\partial {C}^{*}}{\partial {X}^{*}}+{V}^{*}\frac{\partial {C}^{*}}{\partial {Y}^{*}}+{\delta }_{c}\left({{U}^{*}}^{2}\frac{{\partial }^{2}{C}^{*}}{\partial {{X}^{*}}^{2}}+{U}^{*}\frac{\partial {C}^{*}}{\partial {X}^{*}}\frac{\partial {U}^{*}}{\partial {X}^{*}}+{U}^{*}\frac{\partial {V}^{*}}{\partial {X}^{*}}\frac{\partial {C}^{*}}{\partial {Y}^{*}}+2{U}^{*}{V}^{*}\frac{{\partial }^{2}{C}^{*}}{\partial {X}^{*}\partial {Y}^{*}}+{{V}^{*}}^{2}\frac{{\partial }^{2}{C}^{*}}{\partial {{Y}^{*}}^{2}}\right.$$

26 $$\left.+{V}^{*}\frac{\partial {C}^{*}}{\partial {Y}^{*}}\frac{\partial {V}^{*}}{\partial {Y}^{*}}+{V}^{*}\frac{\partial {U}^{*}}{\partial {Y}^{*}}\frac{\partial {C}^{*}}{\partial {X}^{*}}\right)=\frac{1}{Sc}\left(\frac{{\partial }^{2}{C}^{*}}{\partial {{X}^{*}}^{2}}+\frac{{\partial }^{2}{C}^{*}}{\partial {{Y}^{*}}^{2}}\right)-{\Gamma }^{*}{C}^{*},$$

Equations (20) transform into the following form,

$$\frac{\partial {C}^{*}}{\partial {Y}^{*}}=-{Bi}_{2}\left(1-{C}^{*}\right), {V}^{*}=0, \frac{\partial {T}^{*}}{\partial {Y}^{*}}=-{Bi}_{1}\left(1-{T}^{*}\right), {U}^{*}={X}^{*} \mathrm{at} {Y}^{*}=0,$$

27 $${C}^{*}\to 0, {U}^{*}=\frac{{c}^{*}}{{b}^{*}}{X}^{*}+ \frac{{a}^{*}}{{b}^{*}} {Y}^{*}, {T}^{*}\to \infty , \mathrm{as\, } {Y}^{*}\to \infty .$$

where,

$$Rd=\frac{4{\sigma }_{1}{\widehat{T}}_{\infty }^{3}}{{\widehat{k}}_{1}\widehat{k}},\, {\beta }^{*}=\widehat{\lambda }{b}^{*},\, \zeta =\frac{\pi }{\widehat{\delta }}{\left(\frac{\widehat{\vartheta }}{{b}^{*}}\right)}^{1/2}, \,Pr=\frac{\widehat{\mu }{\widehat{C}}_{\widehat{P}}}{\widehat{k}},\, M=\frac{\widehat{\sigma }{\widehat{B}}_{0}^{2}}{\widehat{\rho }{b}^{*}},\, A=\frac{\pi {\widehat{j}}_{0}{\widehat{M}}_{0}}{8\widehat{\rho }{b}^{*}{(\widehat{\vartheta }{b}^{*})}^{1/2}} ,$$

28 $${\delta }_{e}={\widehat{\gamma }}_{E}{b}^{*}, \,Sc=\frac{\widehat{\vartheta }}{\widehat{D}},\, {Bi}_{2}=\frac{{\widehat{h}}_{2}}{\widehat{D}}{\left(\frac{\widehat{\vartheta }}{{b}^{*}}\right)}^{1/2}, \,{\delta }_{c}={\widehat{\gamma }}_{C}{b}^{*},\, {\Gamma }^{*}=\frac{\widehat{\xi }\left({\widehat{C}}_{w}-{\widehat{C}}_{\infty }\right)}{{b}^{*}} , {Bi}_{1}=\frac{{\widehat{h}}_{1}}{\widehat{k}}{\left(\frac{\widehat{\vartheta }}{{b}^{*}}\right)}^{1/2}.$$

Now we define the velocity components in terms of stream function as follows^2^,

29 $${V}^{*}=-\frac{\partial \psi }{\partial {X}^{*}}, {U}^{*}=\frac{\partial \psi }{\partial {Y}^{*}},$$

Now using^2^

30 $$\psi ={X}^{*}F\left({Y}^{*}\right)+H\left({Y}^{*}\right), {T}^{*}=\theta \left({Y}^{*}\right), {C}^{*}=\Phi ({Y}^{*})$$

Now utilize Eqs. (29) and (30) into Eqs. (23), (24), (25) and (26). After eliminating the pressure terms and comparing the coefficients of ${{X}^{*}}^{0}$ and ${{X}^{*}}^{1}$, we get the following nonlinear equations.

$$\frac{{d}^{4}F}{d{{Y}^{*}}^{4}}+F\left({Y}^{*}\right)\frac{{d}^{3}F}{d{{Y}^{*}}^{3}}+{\left(\frac{{d}^{2}F}{d{{Y}^{*}}^{2}}\right)}^{2}-2\frac{dF}{d{Y}^{*}}\frac{{d}^{2}F}{d{{Y}^{*}}^{2}}+{\beta }^{*}\left(2\left({\left(\frac{dF}{d{Y}^{*}}\right)}^{2}\frac{{d}^{2}F}{d{{Y}^{*}}^{2}}+F\left({Y}^{*}\right){\left(\frac{{d}^{2}F}{d{{Y}^{*}}^{2}}\right)}^{2}\right)\right.$$

31 $$\left.-{\left(F\left({Y}^{*}\right)\right)}^{2}\frac{{d}^{4}F}{d{{Y}^{*}}^{4}}\right)-M\frac{{d}^{2}F}{d{{Y}^{*}}^{2}}=0,$$

$$\frac{{d}^{4}H}{d{{Y}^{*}}^{4}}+F\left({Y}^{*}\right)\frac{{d}^{3}H}{d{{Y}^{*}}^{3}}-\frac{{d}^{2}F}{d{{Y}^{*}}^{2}}\frac{dH}{d{Y}^{*}}+{\beta }^{*}\left(2\frac{dF}{d{Y}^{*}}\right.\frac{{d}^{2}F}{d{{Y}^{*}}^{2}}\frac{dH}{d{Y}^{*}}+2F\left({Y}^{*}\right)\frac{dH}{d{Y}^{*}}\frac{{d}^{3}F}{d{{Y}^{*}}^{3}}+2F\left({Y}^{*}\right)\frac{{d}^{2}F}{d{{Y}^{*}}^{2}}\frac{{d}^{2}H}{d{{Y}^{*}}^{2}}$$

32 $$\left.-F\left({Y}^{*}\right)\frac{dF}{d{Y}^{*}}\frac{{d}^{3}H}{d{{Y}^{*}}^{3}}-{\left(F\left({Y}^{*}\right)\right)}^{2}\frac{{d}^{4}H}{d{{Y}^{*}}^{4}}\right)-M\frac{{d}^{2}H}{d{{Y}^{*}}^{2}}-A\zeta \mathrm{exp}\left(-\zeta {Y}^{*}\right)=0,$$

33 $$\left(1+\frac{4}{3}Rd\right)\frac{{d}^{2}\theta }{d{{Y}^{*}}^{2}}+\mathrm{Pr}\left(F\left({Y}^{*}\right)\frac{d\theta }{d{Y}^{*}}-{\delta }_{e}\left(F\left({Y}^{*}\right)\frac{dF}{d{Y}^{*}}\frac{d\theta }{d{Y}^{*}}+{\left(F\left({Y}^{*}\right)\right)}^{2}\frac{{d}^{2}\theta }{d{{Y}^{*}}^{2}}\right)\right)=0,$$

34 $$\frac{{d}^{2}\Phi }{d{{Y}^{*}}^{2}}+\mathrm{Sc}\left(F\left({Y}^{*}\right)\frac{d\Phi }{d{Y}^{*}}-{\delta }_{c}\left(F\left({Y}^{*}\right)\frac{dF}{d{Y}^{*}}\frac{d\Phi }{d{Y}^{*}}+{\left(F\left({Y}^{*}\right)\right)}^{2}\frac{{d}^{2}\Phi }{d{{Y}^{*}}^{2}}\right)-{\Gamma }^{*}\Phi \left({Y}^{*}\right)\right)=0,$$

Equation (27) becomes,

$$\frac{dF}{d{Y}^{*}}=1, \frac{d\Phi }{d{Y}^{*}}={-Bi}_{2}\left(1-\Phi \left({Y}^{*}\right)\right), \frac{dH}{d{Y}^{*}}=0, F\left({Y}^{*}\right)=0, \frac{d\theta }{d{Y}^{*}}={-Bi}_{1}\left(1-\theta \left({Y}^{*}\right)\right) \mathrm{at} {Y}^{*}=0,$$

35 $$\frac{dF}{d{Y}^{*}}=\frac{{c}^{*}}{{b}^{*}}, \frac{dH}{d{Y}^{*}}=\Lambda {Y}^{*}, \frac{{d}^{2}H}{d{{Y}^{*}}^{2}}=\Lambda , \theta \left({Y}^{*}\right)\to 0, \Phi \left({Y}^{*}\right)\to 0 \mathrm{as} {Y}^{*}\to \infty .$$

After performing the integration to Eqs. (31) and (32), we obtain

36 $$\frac{{d}^{3}F}{d{{Y}^{*}}^{3}}+F\left({Y}^{*}\right)\frac{{d}^{2}F}{d{{Y}^{*}}^{2}}-{\left(\frac{dF}{d{Y}^{*}}\right)}^{2}+{\beta }^{*}\left(2F\left({Y}^{*}\right)\frac{dF}{d{Y}^{*}}\frac{{d}^{2}F}{d{{Y}^{*}}^{2}}-{\left(F\left({Y}^{*}\right)\right)}^{2}\frac{{d}^{3}F}{d{{Y}^{*}}^{3}}\right)-M\frac{dF}{d{Y}^{*}}+{C}_{1}=0,$$

$$\frac{{d}^{3}H}{d{{Y}^{*}}^{3}}+F\left({Y}^{*}\right)\frac{{d}^{2}H}{d{{Y}^{*}}^{2}}-\frac{dF}{d{Y}^{*}}\frac{dH}{d{Y}^{*}}+{\beta }^{*}\left(2F\left({Y}^{*}\right)\frac{dH}{d{Y}^{*}}\frac{{d}^{2}F}{d{{Y}^{*}}^{2}}-{\left(F\left({Y}^{*}\right)\right)}^{2}\frac{{d}^{3}H}{d{{Y}^{*}}^{3}}\right)$$

37 $$-M\frac{dH}{d{Y}^{*}}+A{e}^{-\zeta {Y}^{*}}+{C}_{2}=0,$$

Now using condition at infinity given in Eq. (35), we have

38 $$F\left({Y}^{*}\right)=\frac{{c}^{*}}{{b}^{*}}{Y}^{*}+\omega , \frac{dF}{d{Y}^{*}}=\frac{{c}^{*}}{{b}^{*}}, \frac{dH}{d{Y}^{*}}=\Lambda {Y}^{*}, \frac{{d}^{2}H}{d{{Y}^{*}}^{2}}=\Lambda ,$$

Using Eq. (38) into Eqs. (36) and (37), we have

39 $${C}_{1}=\frac{{c}^{*}}{{b}^{*}}\left(\frac{{c}^{*}}{{b}^{*}}+M\right), {C}_{2}=-\omega \Lambda ,$$

Equations (36), (37) and (39) becomes,

$$\frac{{d}^{3}F}{d{{Y}^{*}}^{3}}+F\left({Y}^{*}\right)\frac{{d}^{2}F}{d{{Y}^{*}}^{2}}-{\left(\frac{dF}{d{Y}^{*}}\right)}^{2}+{\beta }^{*}\left(2F\left({Y}^{*}\right)\frac{dF}{d{Y}^{*}}\frac{{d}^{2}F}{d{{Y}^{*}}^{2}}-{\left(F\left({Y}^{*}\right)\right)}^{2}\frac{{d}^{3}F}{d{{Y}^{*}}^{3}}\right)$$

40 $$+M\left(\frac{{c}^{*}}{{b}^{*}}-\frac{dF}{d{Y}^{*}}\right)+{\left(\frac{{c}^{*}}{{b}^{*}}\right)}^{2}=0,$$

$$\frac{{d}^{3}H}{d{{Y}^{*}}^{3}}+F\left({Y}^{*}\right)\frac{{d}^{2}H}{d{{Y}^{*}}^{2}}-\frac{dF}{d{Y}^{*}}\frac{dH}{d{Y}^{*}}+{\beta }^{*}\left(2F\left({Y}^{*}\right)\frac{dH}{d{Y}^{*}}\frac{{d}^{2}F}{d{{Y}^{*}}^{2}}-{\left(F\left({Y}^{*}\right)\right)}^{2}\frac{{d}^{3}H}{d{{Y}^{*}}^{3}}\right)$$

41 $$-M\frac{dH}{d{Y}^{*}}+A{e}^{-\zeta {Y}^{*}}-\omega \Lambda =0,$$

Now introducing $\frac{dH}{d{Y}^{*}}=\Lambda G\left({Y}^{*}\right),$ Eq. (41) becomes

$$\frac{{d}^{2}G}{d{{Y}^{*}}^{2}}+F\left({Y}^{*}\right)\frac{dG}{d{Y}^{*}}-\frac{dF}{d{Y}^{*}}G\left({Y}^{*}\right)+{\beta }^{*}\left(2F\left({Y}^{*}\right)G\left({Y}^{*}\right)\frac{{d}^{2}F}{d{{Y}^{*}}^{2}}-{\left(F\left({Y}^{*}\right)\right)}^{2}\frac{{d}^{2}G}{d{{Y}^{*}}^{2}}\right)$$

42 $$-MG\left({Y}^{*}\right)+{A}^{*}{e}^{-\zeta {Y}^{*}}-\omega =0,$$

The relevant conditions are,

43 $$G\left(0\right)=0, \frac{dG(\infty )}{d{Y}^{*}}=1.$$

According to the engineering point of view, the physical quantities of interest (Sherwood number, skin friction coefficient, Nusselt number) are very significant. For the ongoing flow phenomenon, these important quantities have the following form^2,32^,

44 $$Sh=\frac{\widehat{X}{\widehat{q}}_{m}}{\widehat{D}\left({\widehat{C}}_{w}-{\widehat{C}}_{\infty }\right)}, {C}_{f}=\frac{{\widehat{\tau }}_{w}}{\widehat{\rho }{{\widehat{U}}_{w}}^{2}}, Nu=\frac{\widehat{X}{(\widehat{q}}_{w}+{\widehat{q}}_{r})}{\widehat{k}\left({\widehat{T}}_{w}-{\widehat{T}}_{\infty }\right)},$$

Here Ref.^32^,

45 $${\widehat{q}}_{m}=-\widehat{D}{\left.\frac{\partial \widehat{C}}{\partial \widehat{Y}}\right|}_{\widehat{Y}=0}, {\widehat{\tau }}_{w}=\widehat{\mu }\left(1+{\beta }^{*}\right){\left.\frac{\partial \widehat{U}}{\partial \widehat{Y}}\right|}_{\widehat{Y}=0}, {\widehat{q}}_{w}=-\widehat{k}{\left.\frac{\partial \widehat{T}}{\partial \widehat{Y}}\right|}_{\widehat{Y}=0},$$

After using Eqs. (21), (30), and (45) in Eq. (44), we have

$${Sh}_{{X}^{*}}{\left({Re}_{{X}^{*}}\right)}^{-0.5}=-\frac{d\Phi \left(0\right)}{d{Y}^{*}}, {C}_{f}{\left({Re}_{{X}^{*}}\right)}^{0.5}=\left(1+{\beta }^{*}\right)\frac{{d}^{2}F\left(0\right)}{d{{Y}^{*}}^{2}},$$

46 $${Nu}_{{X}^{*}}{\left({Re}_{{X}^{*}}\right)}^{-0.5}=-\left(1+\frac{4}{3}Rd\right)\frac{d\theta \left(0\right)}{d{Y}^{*}}, {Re}_{{X}^{*}}=\frac{{X}^{*}{\widehat{U}}_{w}}{\widehat{\vartheta }}.$$

## Result and explanation

**Figure 2:**
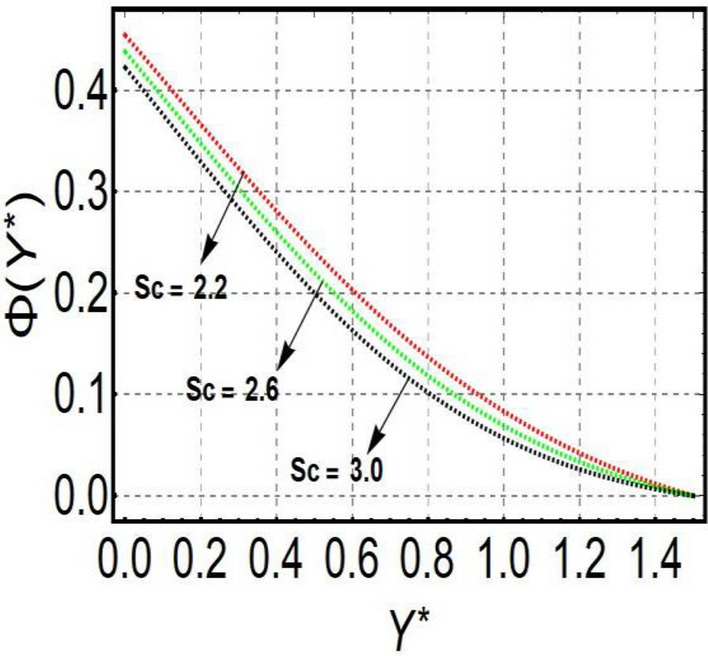
Curve of $\Phi \left({Y}^{*}\right)$ relative to $Sc$.

**Figure 3:**
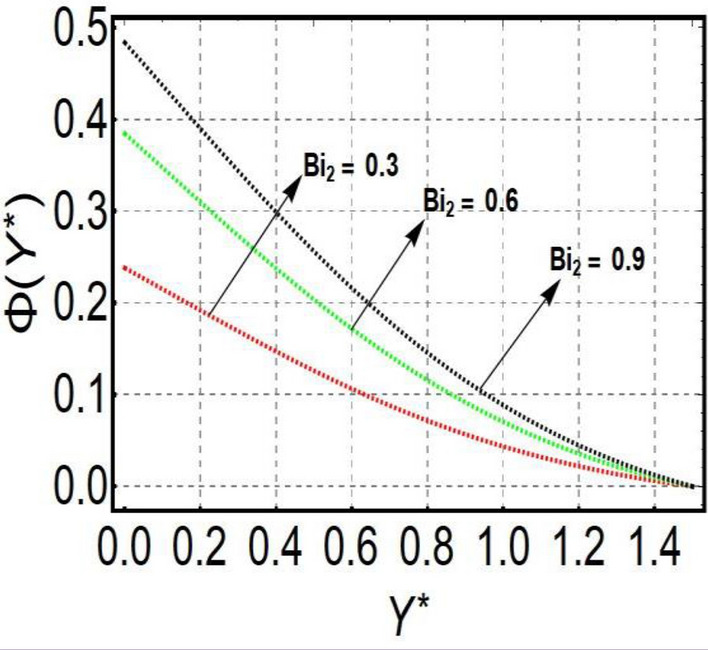
Curve of $\Phi \left({Y}^{*}\right)$ relative to ${Bi}_{2}$.

**Figure 4:**
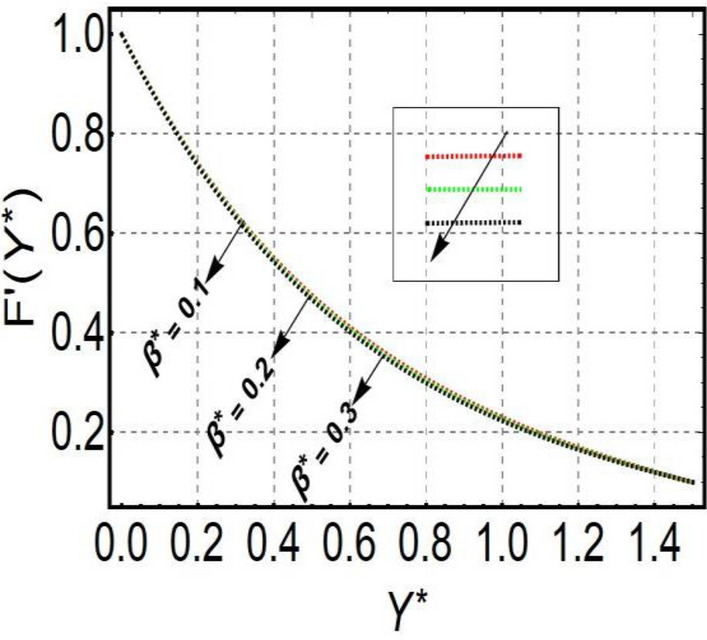
Curve of $F\mathrm{^{\prime}}\left({Y}^{*}\right)$ relative to ${\beta }^{*}$.

**Figure 5:**
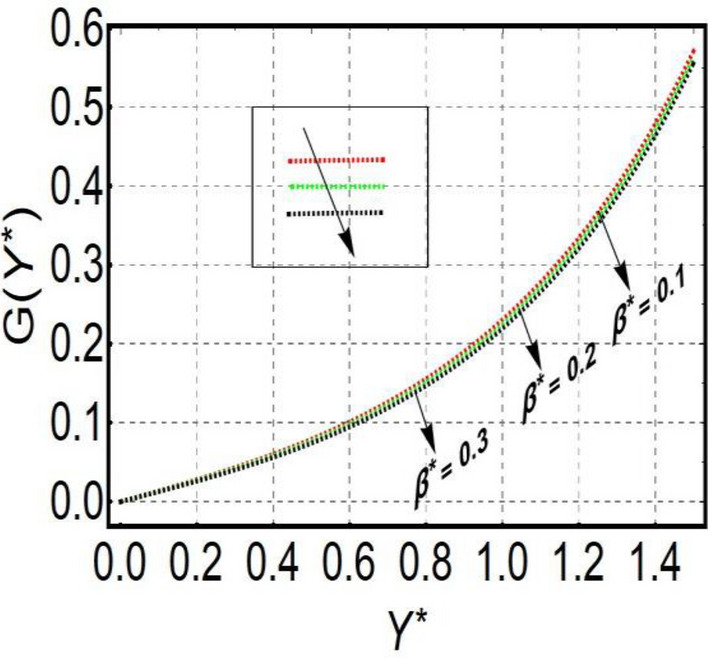
Curve of $G \left({Y}^{*}\right)$ relative to ${\beta }^{*}$.

**Figure 6:**
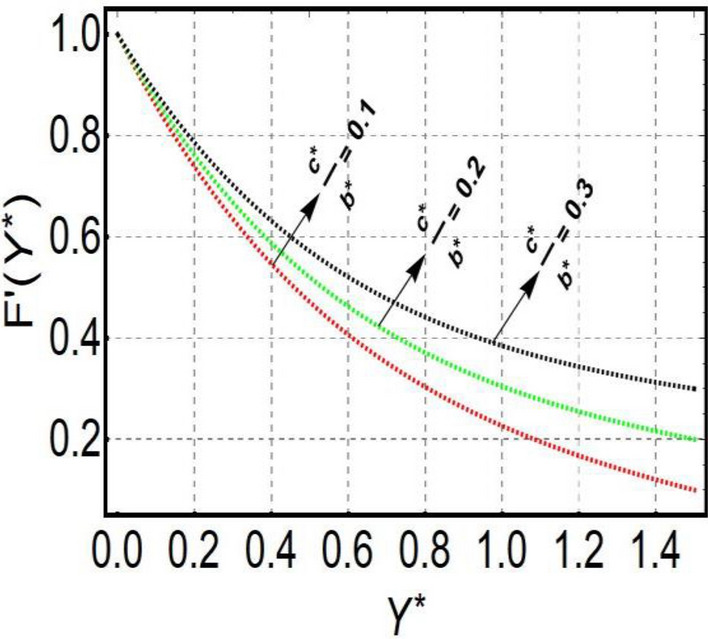
Curve of $F\mathrm{^{\prime}}\left({Y}^{*}\right)$ relative to $\frac{{c}^{*}}{{b}^{*}}$.

**Figure 7:**
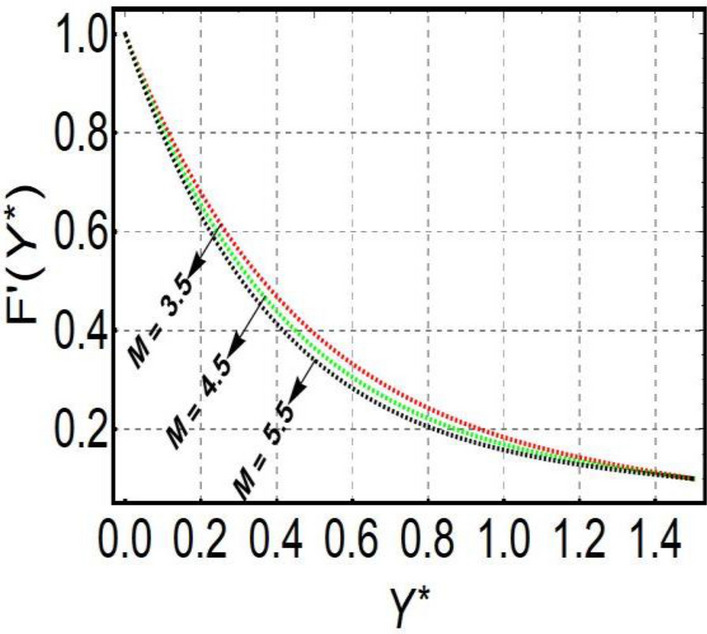
Curve of $F\mathrm{^{\prime}}\left({Y}^{*}\right)$ relative to $M$.

**Figure 8:**
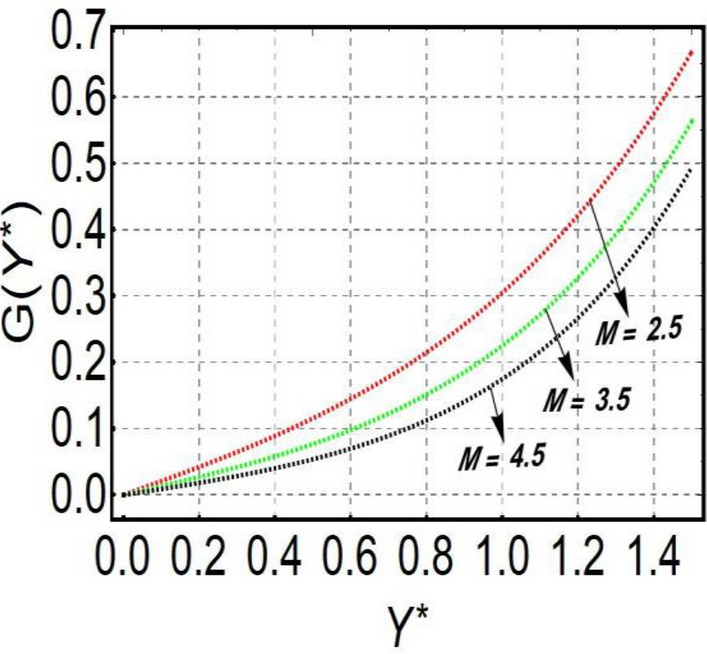
Curve of $G\left({Y}^{*}\right)$ relative to $M$.

**Figure 9:**
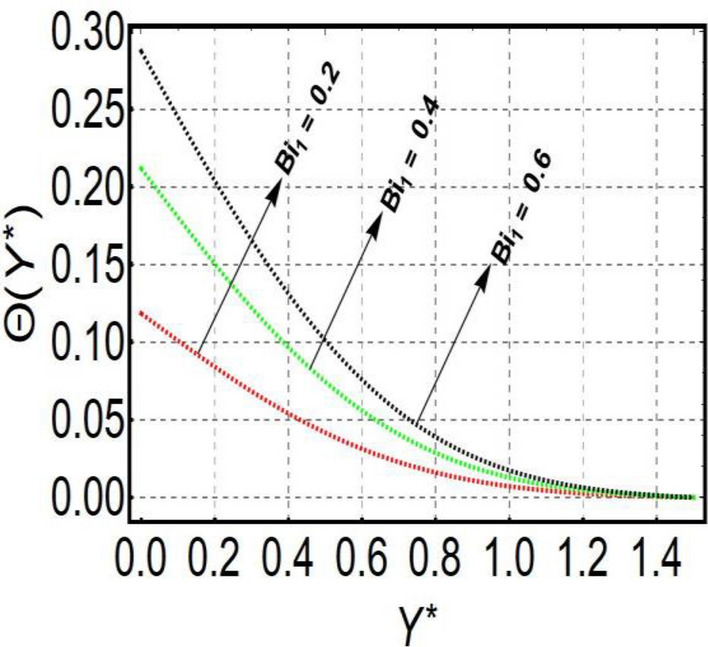
Curve of $\theta \left({Y}^{*}\right)$ relative to ${Bi}_{1}$.

**Figure 10:**
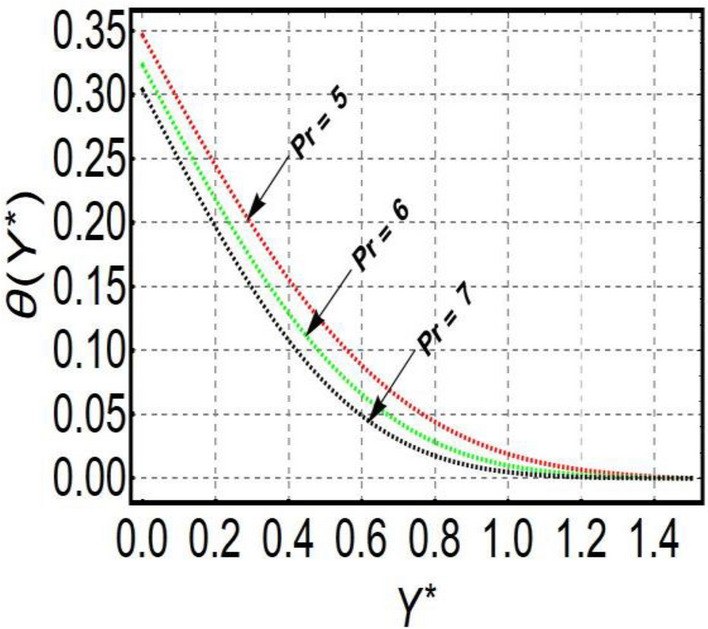
Curve of $\theta \left({Y}^{*}\right)$ relative to $Pr$.

**Figure 11:**
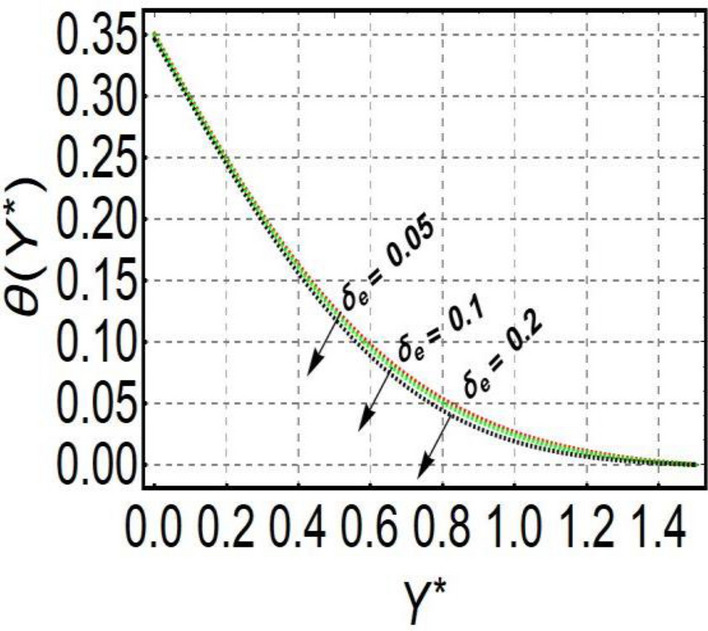
Curve of $\theta \left({Y}^{*}\right)$ relative to ${\delta }_{e}$.

**Figure 12:**
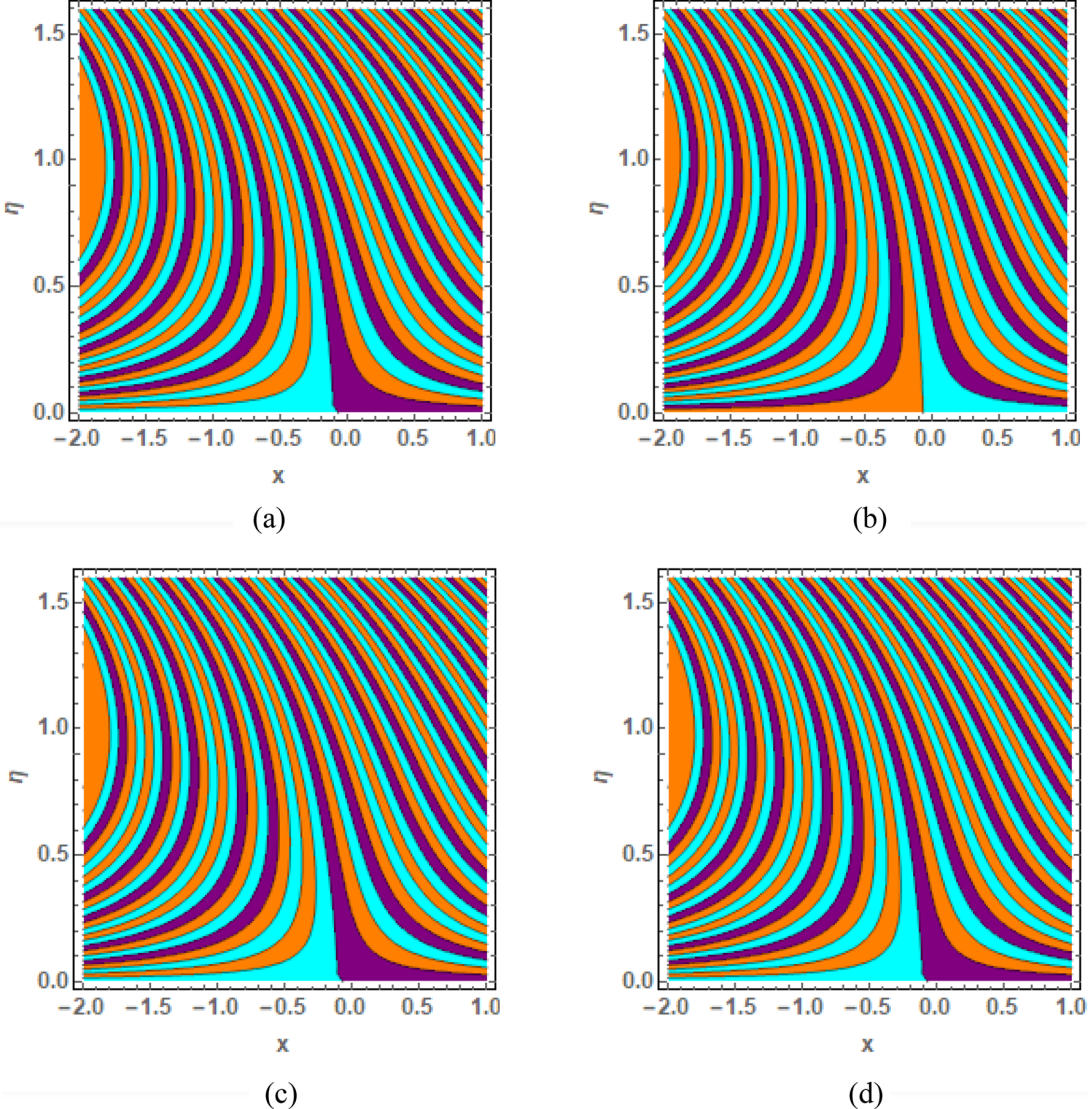
Streamline pattern by varying *M*. (**a**) *M* = 2.5, (**b**) *M* = 3.5, (**c**) *M* = 4.5, (**d**) *M* = 5.5.

**Figure 13:**
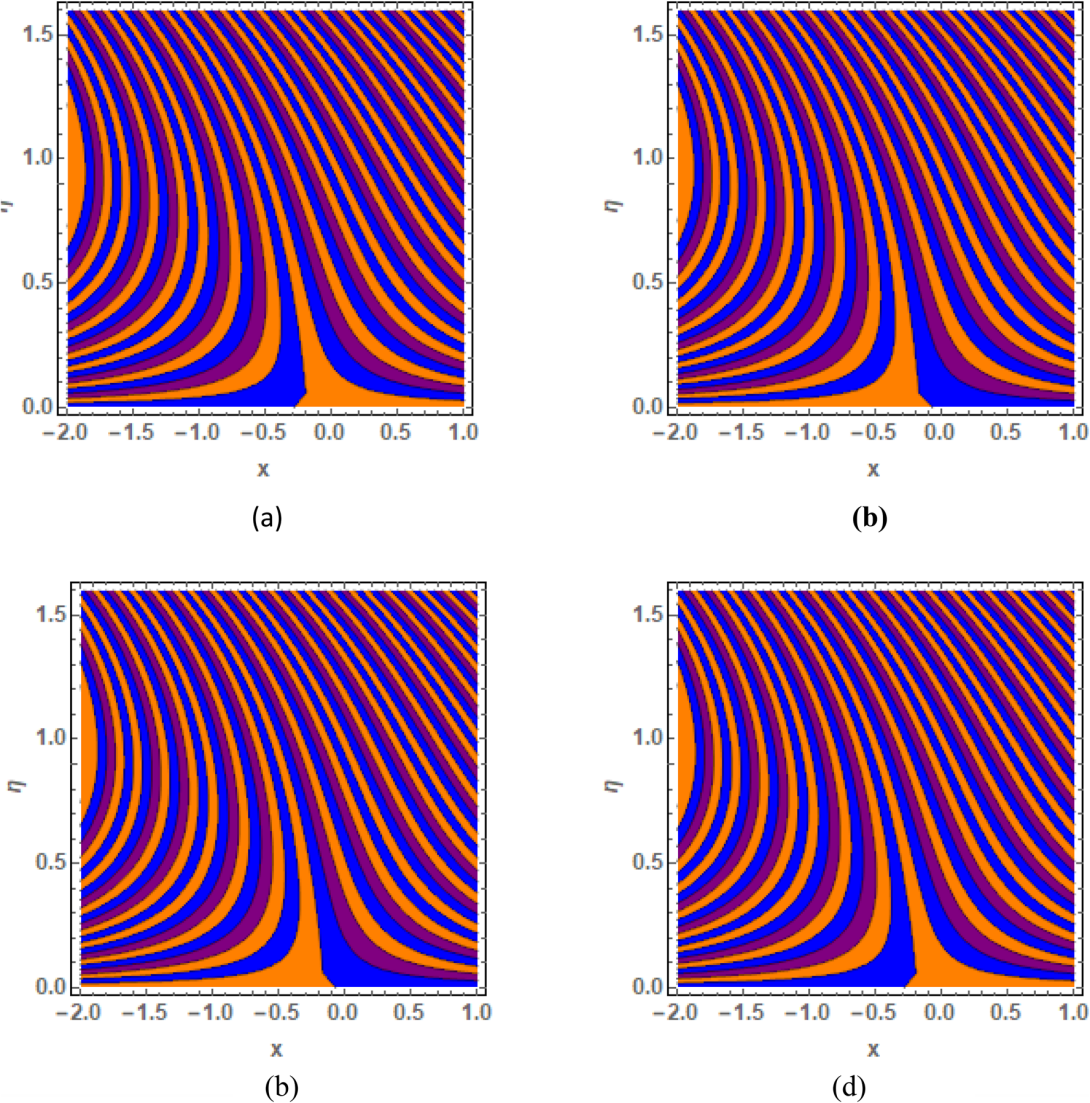
Streamline pattern by varying $\beta =0.1$, $\beta =.4, \beta =.5 ,\beta =1$.

**Figure 14:**
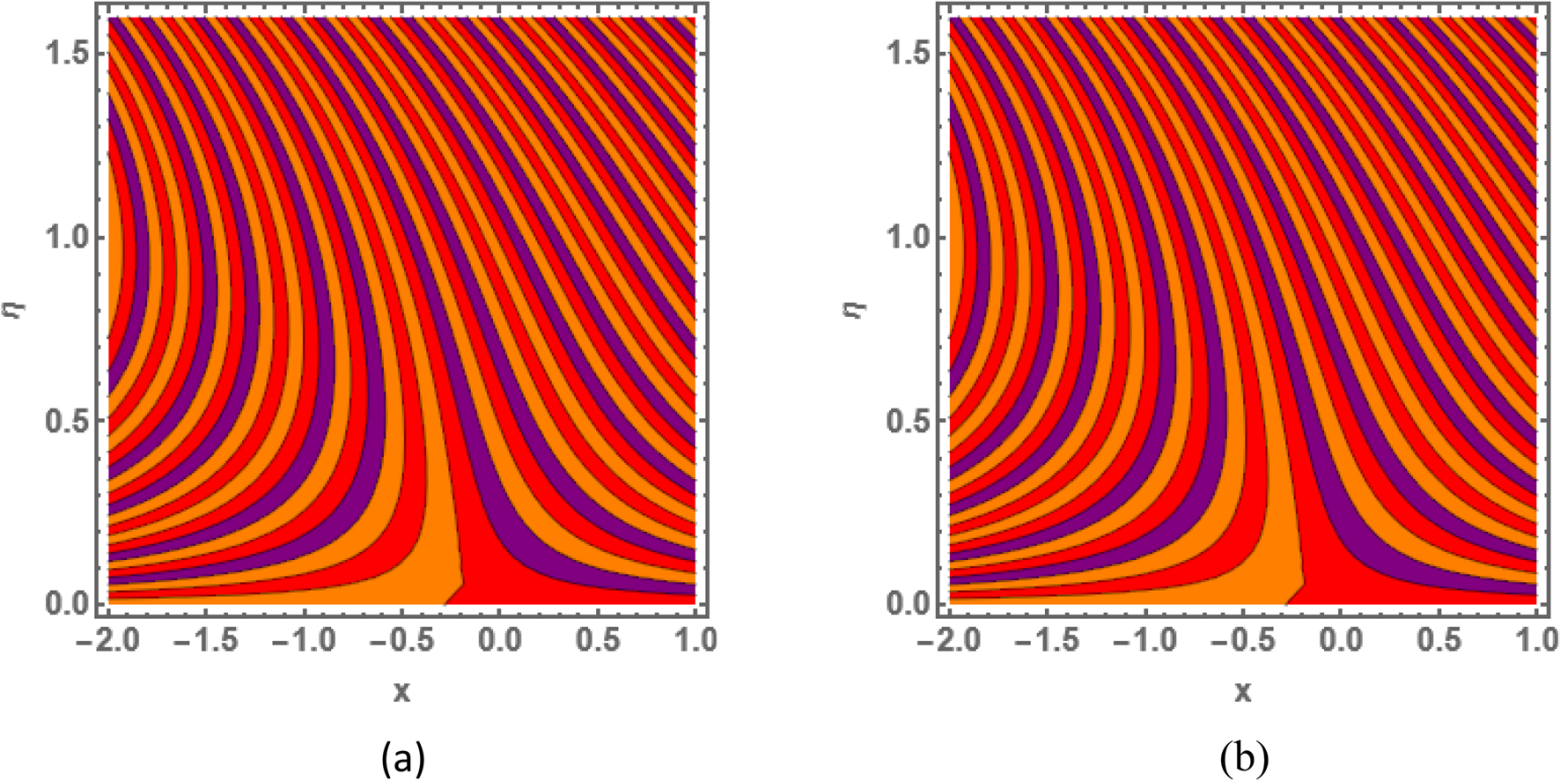
Streamline pattern by varying $\frac{{c}^{*}}{{b}^{*}}$. (**a**) $\frac{{c}^{*}}{{b}^{*}}$= 0.1, (**b**) $\frac{{c}^{*}}{{b}^{*}}$ = 0.3.

**Figure 15:**
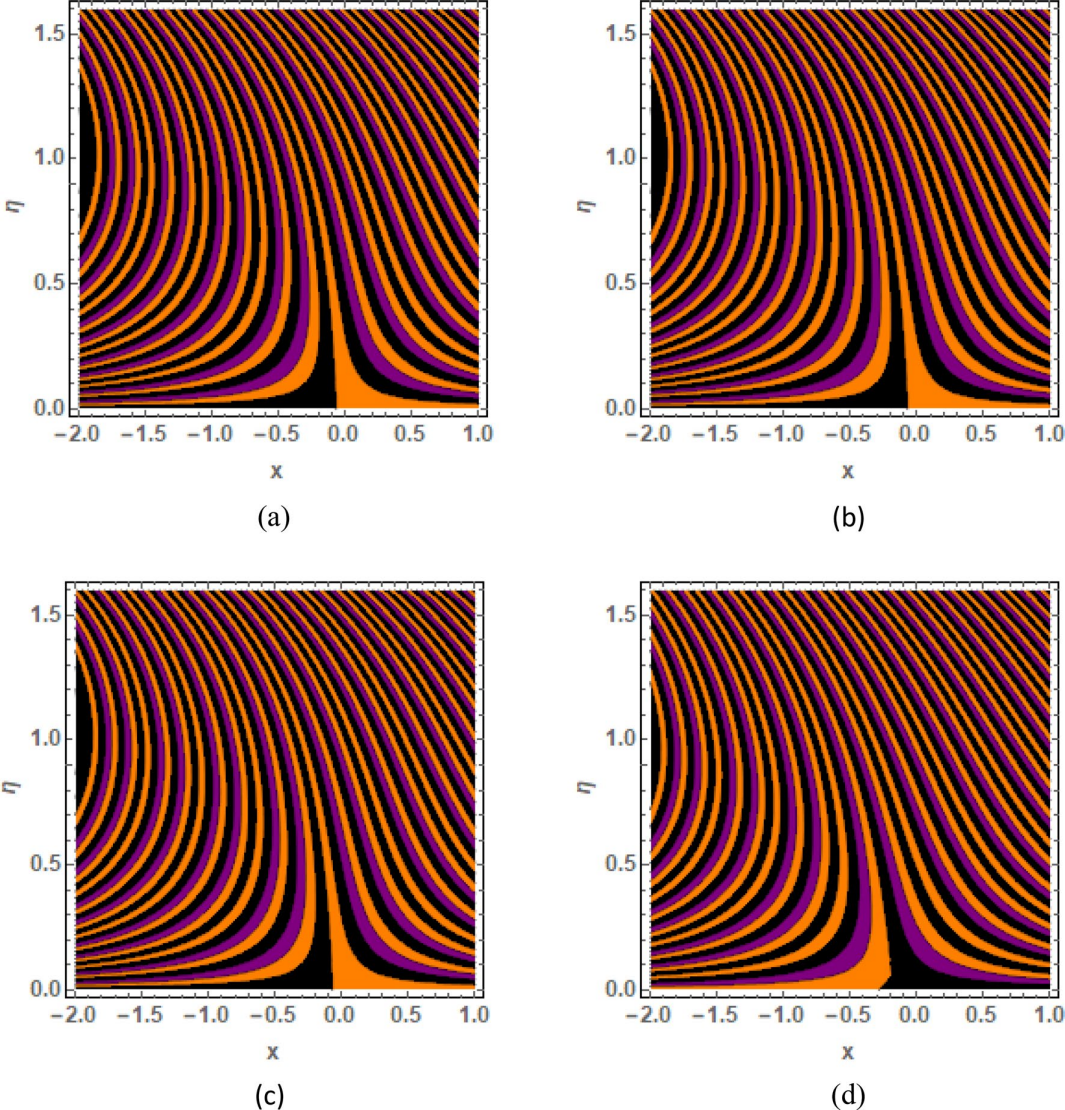
Streamline pattern by varying Pr.(**a**) *Pr* = 2.5, (**b**) *Pr* = 3.5, (**c**) *Pr* = 4.5, (**d**)*Pr* = 5.5.

This section is prepared to numerically contemplate the nonlinear system of ordinary differential equations through the symbolic package Mathematica. For the validation and accuracy of the current analysis, a comparative study between the previous and current outcomes is carried out in Table 1. This Table demonstrates that a good relationship is established between the current and previous results. Table 2 is organized to numerically examine the consequences of different parameters on the physical quantities. From this Table, it is noticed that both the quantities (Nusselt number and Sherwood number) minimize with the improved Deborah number, but the quantity of skin friction coefficient enhanced for this parameter. The impact of the stretching ratio parameter on all these quantities is opposite as compared to the Deborah number. Both the quantities of the Nusselt number and Sherwood number deteriorate relative to the thermal and concentration relaxation parameters respectively. Various features of the flow phenomenon corresponding to the emerging pertinent parameters are graphically visualized. Figure 2 portrays the behavior of concentration distribution subject to the accelerating values of Schmidt number. It is examined that the improved Schmidt number deteriorates the profile of the concentration. The physical reason for this phenomenon is that the mass diffusivity is inversely related to the Schmidt number. The mass diffusivity diminishes with the enhanced Schmidt number. Consequently, the curve of the concentration distribution exhibits a descending nature. The consequence of the concentration Biot number on the profile of the concentration is revealed in Fig. 3. This graphic exhibits that the higher magnitude of the concentration Biot number develops an augmentation in the concentration distribution. Physically, the concentration Biot number is directly related to the mass transfer coefficient. The mass transfer rate is enhanced with the improved Biot number which further escalates the concentration behavior. Figures 4 and 5 are sketched to explore the nature of both axial and tangential velocities relative to the Deborah number. These graphics disclose the diminishing behavior of both velocities with the accelerating amount of the Deborah number. This phenomenon happens because the fluid’s viscosity is enhanced with the improved Deborah number. The higher intensity of the fluid viscosity generates resistance in the movement of the fluid and declines the velocity curve. Figure 6 is prepared to scrutinize the flow field of axial velocity influenced by the velocity ratio parameter. This figure reveals that the increasing impact of the velocity ratio parameter produces an escalation in the axial velocity field. The deteriorating nature of both velocities (axial and tangential) corresponding to the stronger magnetic field is manifested in Figs. 7 and 8. Physically, with the contribution of electric and magnetic fields, a resistive Lorentz force is generated. This resistive force functions in the reverse direction of the fluid flow and reduces fluid transport. Accordingly, the curves of axial and tangential velocities demonstrate the descending nature. The field of the fluid temperature in response to the improved thermal Biot number is depicted in Fig. 9. Due to the relation of the thermal Biot number with the heat transfer coefficient, the heat transport rate enhances with the greater intensity of the thermal Biot number. As a result, the temperature distribution portrays the escalating nature. The relation between the temperature field and the Prandtl number is manifested in Fig. 10. With a greater amount of the Prandtl number, there exists a reduction in the curve of the temperature. The reason is the inverse connection of thermal conductivity with the Prandtl number. The fluid’s thermal conductivity becomes lower with the larger Prandtl number. As a result, the rate of heat distribution is minimized, and the thermal field illustrates the dwindling behavior. The purpose of Fig. 11 is to investigate the nature of the temperature distribution compared to the thermal relaxation parameter. The higher magnitude of the thermal relaxation parameter depletes the field of the fluid temperature. With the greater thermal relaxation time parameter, the particles of the fluid consume more time to accomplish the process of heat transfer to its neighboring particles. Accordingly, the fluid temperature profile becomes diminishes. Figures 12, 13, 14 and 15 depict the streamline pattern for varying values of the magnetic parameter, Deborah number and velocity ratio parameter. Streamlines predict the path of suspended imaginary particles in the liquid and passed along with it. We consider steady flow where the streamlines are fixed meanwhile the fluid is in motion. On oblique stagnation is clearly evident through stream line graph.A Stagnation point can be seen at center 0 at some parameters.Stream lines show that how the follow disperesed around the staganation point. Figure 12a–d depicts the streamline pattern by varying magnetic parameter (M). By changing the magnetic parameter M from 2.5 to 5.5, the stagnation point moves to the right of the flow pattern. As described earlier, with the contribution of electric and magnetic fields, a resistive Lorentz force is generated. This resistive force functions in the reverse direction of the fluid flow and reduces fluid transport. Hence, the stagnation point moves to axial location at zero. Figure 13a–d depicts the streamline pattern by changing Deborah number. Enhancement in Deborah number from 0.1 to 0.3 leads to negligible impact on the stagnation point and streamline pattern. Figure 14a–b depicts the streamline pattern by varying velocities ratio. Enhancement in velocities ratio from 0.1 to 0.3 leads to negligible impact on the stagnation point and streamline pattern. Figure 15a–d depicts the streamline pattern by changing Prandtle number (Pr). Enhancement in Prandtle number from 1 to 4 leads impact on the stagnation point and streamline pattern are showed by changing Prandtle number.

**Table 1:** Comparison of $-\left(1+\frac{4}{3}Rd\right)\frac{d\uptheta \left(0\right)}{d{Y}^{*}}$ for distinct values of $Pr$ when $\frac{{c}^{*}}{{b}^{*}}=0.1, {Bi}_{1}=0.1 ,Rd=Sc=1.$

$Pr$	Present results	Ghaffari et al.^2^
0.7	0.1758	0.1681
1	0.1766	0.1768
10	0.1879	0.2164
50	0.1948	0.2250

**Table 2:** Numerical values of physical quantities subject to the various values of parameters.

$\frac{{c}^{*}}{{b}^{*}}$	${\beta }^{*}$	${\delta }_{e}$	${\delta }_{c}$	$M$	${Sh}_{{X}^{*}}{\left({Re}_{{X}^{*}}\right)}^{-0.5}$	$-{C}_{f}{\left({Re}_{{X}^{*}}\right)}^{0.5}$	${Nu}_{{X}^{*}}{\left({Re}_{{X}^{*}}\right)}^{-0.5}$
0.1	0.2	0.2	0.2	0.5	0.2644	1.5035	0.3117
0.2					0.2651	1.3870	0.3129
0.3					0.2659	1.2576	0.3142
	0.3				0.2643	1.6503	0.3116
	0.4				0.2642	1.8004	0.3114
	0.5				0.2641	1.9540	0.3113
		0.1			0.2644	1.5035	0.3121
		0.2			0.2644	1.5035	0.3117
		0.3			0.2644	1.5035	0.3113
			0.1		0.2676	1.5035	0.3117
			0.2		0.2644	1.5035	0.3117
			0.3		0.2609	1.5035	0.3117
				0.5	0.3949	1.5035	0.4827
				0.7	0.3944	1.5722	0.4819
				0.9	0.3938	1.6389	0.4810

## Concluding remarks

An exploration of the hydromagnetic steady flow of a Maxwell rate-type fluid near an oblique stagnation point flow is carried out. A convective stretched Riga plate is considered to be the source of the radiative flow phenomenon. The two-dimensional flow problem is analyzed with the involvement of Cattaneo-Christov theory and the physical effects of the chemical reaction. The important results of this study are illustrated through the following points.

- The concentration distribution is the decreasing function of the Schmidt number.
- The improved concentration Biot number escalates the field of the concentration distribution.
- Both the velocities (tangential and axial) portray the dwindling behavior corresponding to the Deborah number and magnetic field.
- The ascending behavior of axial velocity is examined with the influence of the velocity ratio parameter.
- With the increment of the Prandtl number and thermal relaxation parameter, the temperature curve goes downward.
- A significant enhancement of heat transport rate is observed relative to the larger intensity of the thermal Biot number.
- The stretching ratio parameter enhances the heat and mass transfer rates.
- The current flow problem can be examined for numerous non-Newtonian fluids with the significance of nonlinear thermal radiation, inclined magnetic field, and partial slip effects. Moreover, the current flow phenomenon can be analyzed with the implementation of the two-phase Buongiorno model.

## Data Availability

The authors states that all the files are provided in the paper no hidden file is required however if journal required any further data from us we will provide and the corresponding author is responsible to provide to the journal.

## List of symbols

${\varvec{V}}$: Velocity field vector
${Bi}_{2}$: Concentration Biot number
$$\widehat{P}$$: Fluid pressure
$$\widehat{\mu }$$: Dynamic viscosity
$\widehat{\xi }$: Chemical reaction rate constant
$$Rd$$: Radiation parameter
$$\omega ,\Lambda $$: Constants
${\widehat{\gamma }}_{C}$: Solutal relaxation time
${\widehat{q}}_{w}$: Surface heat flux
$${c}^{*},{b}^{*},{a}^{*}$$: Positive constants
${\widehat{C}}_{\widehat{P}}$: Specific heat capacity at constant pressure
${\delta }_{c}$: Concentration relaxation time parameter
${\widehat{j}}_{0}$: Applied current density
${\widehat{T}}_{\infty }$: Ambient fluid temperature
$$\mathbf{J}$$: Mass flux
${\widehat{\gamma }}_{E}$: Thermal relaxation time
${\Gamma }^{*}$: Chemical reaction parameter
$$\widehat{\vartheta }$$: Kinematic viscosity
${\sigma }_{1}$: Stefan-Boltzmann constant
${\widehat{h}}_{1}$: Heat transfer coefficient
${\varvec{B}}$: Magnetic field vector
$$\zeta $$: Dimensional parameter
$$\widehat{\rho }$$: Fluid density
$$M$$: Magnetic parameter
$$\widehat{\sigma }$$: Electrical conductivity
$\widehat{\lambda }$: Relaxation time parameter
$\widehat{\delta }$: Electrodes and magnets width
${\delta }_{e}$: Thermal relaxation time parameter
$A$: Modified Hartmann number
$$\widehat{D}$$: Mass diffusivity
${Re}_{{X}^{*}}$: Local Reynolds number
${\widehat{h}}_{2}$: Mass transfer coefficient
$\widehat{k}$: Fluid thermal conductivity
$$Pr$$: Prandtl number
$$\left(\widehat{U},\widehat{V}\right)$$: Velocity components
$${\beta }^{*}$$: Deborah number
$\overline{{\varvec{\tau}} }$: Extra stress tensor
$${\widehat{M}}_{0}$$: Magnets magnetization
${Bi}_{1}$: Thermal Biot number
$$\mathbf{q}$$: Heat flux
${\widehat{k}}_{1}$: Mean absorption coefficient
${\widehat{C}}_{\infty }$: Ambient fluid concentration
$$\left(\widehat{X},\widehat{Y}\right)$$: Cartesian coordinates
$$Sc$$: Schmidt number
